# Assess suitability of hydroaeroponic culture to establish tripartite symbiosis between different AMF species, beans, and rhizobia

**DOI:** 10.1186/1471-2229-9-73

**Published:** 2009-06-17

**Authors:** Fatma Tajini, Porntip Suriyakup, Hélène Vailhe, Jan Jansa, Jean-Jacques Drevon

**Affiliations:** 1 Institut National de la Recherche Agronomique, UMR1222 Ecologie Fonctionnelle & Biogéochimie des Sols, INRA-IRD-SupAgro, 2 place Viala, 34060 Montpellier Cedex, France; 2 Faculté des Sciences de Gafsa, 2112 Sidi Ahmed Zarroug, Tunisie; 3 Khon Kaen University, Department of Agronomy, Faculty of Agriculture, Khon Kaen 40002, Thailand; 4 ETH Zürich, Plant Sciences, Eschikon 33, CH – 8315 Lindau (ZH), Switzerland

## Abstract

**Background:**

Like other species of the *Phaseoleae *tribe, common bean (*Phaseolus vulgaris *L.) has the potential to establish symbiosis with rhizobia and to fix the atmospheric dinitrogen (N_2_) for its N nutrition. Common bean has also the potential to establish symbiosis with arbuscular mycorrhizal fungi (AMF) that improves the uptake of low mobile nutrients such as phosphorus, from the soil. Both rhizobial and mycorrhizal symbioses can act synergistically in benefits on plant.

**Results:**

The tripartite symbiosis of common bean with rhizobia and arbuscular mycorrhizal fungi (AMF) was assessed in hydroaeroponic culture with common bean (*Phaseolus vulgaris *L.), by comparing the effects of three fungi spp. on growth, nodulation and mycorrhization of the roots under sufficient *versus *deficient P supplies, after transfer from initial sand culture. Although *Glomus intraradices *Schenck & Smith colonized intensely the roots of common bean in both sand and hydroaeroponic cultures, *Gigaspora rosea *Nicolson & Schenck only established well under sand culture conditions, and no root-colonization was found with *Acaulospora mellea *Spain & Schenck under either culture conditions. Interestingly, mycorrhization by *Glomus *was also obtained by contact with mycorrhized *Stylosanthes guianensis *(Aubl.) sw in sand culture under deficient P before transfer into hydroaeroponic culture. The effect of bean genotype on both rhizobial and mycorrhizal symbioses with *Glomus *was subsequently assessed with the common bean recombinant inbreed line 7, 28, 83, 115 and 147, and the cultivar Flamingo. Significant differences among colonization and nodulation of the roots and growth among genotypes were found.

**Conclusion:**

The hydroaeroponic culture is a valuable tool for further scrutinizing the physiological interactions and nutrient partitioning within the tripartite symbiosis.

## Background

Common bean (*Phaseolus vulgaris *L.) is grown generally as a grain-legume in crop rotation or association with cereals, or as snap-bean in horticultural systems. Like other species of the *Phaseoleae *tribe, common bean has the potential to establish symbiosis with rhizobia and to fix the atmospheric dinitrogen (N_2_) for its N nutrition. The amount of N_2 _fixed by legume depends on plant species and cultivars, on rhizobial strains and on the environmental conditions [1]. Common bean has also the potential to establish symbiosis with arbuscular mycorrhizal fungi (AMF) that improves the uptake of low mobile nutrients such as phosphorus, from the soil [2,3]. Both rhizobial and mycorrhizal symbioses can act synergistically on promoting plant growth and fitness [4,5]. This can result in benefits on yield [6,7]. Both symbioses share parts of signalling pathways, indicating intimate interactions between all three partners during co-evolution [8,9].

More P is generally required by legumes, especially when their N nutrition depends upon the rhizobial symbiosis, with up to 20% of total plant P being allocated to nodules. Indeed, nodule biomass is strongly correlated to P availability in plant [10], and is drastically reduced by P deficiency, with major reduction in nodule size [11,12]. However, P requirements for growth and N_2 _fixation differ widely among legume species [13] and among common bean genotypes in particular [14]. Plenchette et al. [15] showed that the calcined clay sprayed with a low-P nutrient solution is a favorable environment for the establishment of the AMF symbiosis and the subsequent stimulation of the plant-growth, although the increase in P concentration of the solution induced a decrease in the rate of mycorrhization that varied with plant spp. Therefore, it is thought that in hydroponic cultures where P in solution is directly available to plants, it is improbable to obtain mycorrhizal effect attributable to P nutrition.

The purpose of the present work was to assess the suitability of hydroaeroponic culture to establish tripartite symbiosis between different AMF species, beans, and rhizobia, and to study the effect of external P supply on symbiosis development and plant response to AMF and rhizobia

## Methods

Two experiments were realized for this work to compare hydroaeroponic and sand cultures: the first with common bean RIL115 and AMF spp. diversity; the second with *Glomus *and common bean genotypic diversity. Both received similar rhizobial inoculation. Thus, the experimental design consisted of randomized complete block with 3 replications. Results were submitted to ANOVA, and comparison of means was achieved by the Duncan's multiple range test (*p *≤ 0.05).

### Plant genotypes and rhizobial inoculation

The common bean (*P. vulgaris*) genotypes used in this study were recombinant inbred lines (RIL) 7, 28, 83, 115, and 147 from the cross of BAT477 and DOR364 (CIAT-INRA cooperation) that are studied by international consortia for their adaptation to low fertility soil, and the cultivar Flamingo (supplied by ESA Mateur, Tunisia, from a collection initially supplied by B. Voyssest from CIAT, Colombia).

Seeds were surface-sterilized with 1.3% calcium hypochloride for 15 min with constant stirring, and subsequently washed with sterile distilled water. They were germinated on 0.8% sterile agar for 3 days at 28°C in the dark, with a germination rate of 80%. Rhizobial inoculation was performed by soaking the seedlings of common bean for 45 min within a freshly prepared suspension of *Rhizobium tropici *CIAT899 containing 10^8 ^bacteria ml^-1^.

### Mycorrhizal inoculation

Thereafter the plants were grown for 2 weeks in 1000 ml pots filled with autoclaved sand-soil mixture (9:1 v:v) recolonized with soil bacteria according to Jansa et al. [16]. The potting substrate was inoculated with AMF in a ratio of 9:1 (v:v) before transfer into hydroaeroponics vats. The inoculum consisted of chopped roots of previous pot cultures planted with leek (*Allium porrum *L.) and grown for 18 months in a glasshouse. Mycorrhizal inoculum contained at least 20 infective propagules of the respective AMF species per gram.

The plants were either inoculated with one of three AMF species, namely *Gigaspora rosea *BEG9 (Nicolson & Schenck), *Glomus intraradices *BEG157 (Schenck & Smith), or *Acaulospora mellea *NM54 (Spain & Schenck).

In order to assess the mycorrhization by contact, some seeds of RIL115 were sown and grown for 2 weeks in contact with mycorrhized *Stylosanthes guianensis *(Aubl.) sw by *Glomus *BEG157, and thereafter transferred into hydroaeroponics vats.

### Tripartite symbiosis culture

After transfer of inoculated seedlings into each pot of sand-soil culture, the plants were grown in a temperature-controlled glasshouse with night/day temperatures of 25/35°C, and a 16 h photoperiod with complementary illumination of 400 μmol photons m^-2 ^s^-1^. After 2 weeks, one plant only was left in the soil-sand substrate. Pots were watered with distilled water every 2 days until harvest, and received once a week the following nutrient solution: macroelements: K_2_SO_4 _(1.25 mM), MgSO_4_.7H_2_O (2.05 mM), CaCl_2 _(3.3 mM); microelements: Fe EDDHA (8.5 μM Fe as sequestrene), H_3_BO_3 _(4.0 μM), MnSO_4 _(6.0 μM), ZnSO_4 _(0.9 μM), CuSO_4 _(1.0 μM), NaMoO_4 _(0.1 μM).

After 2 weeks, two plants from sand culture were transferred into 45 l vats containing 20 plants per vat, and receiving either 75 or 250 μmol KH_2_PO_4 _plant^-1 ^week^-1 ^in addition to the above nutrient solution. This solution was replaced every two weeks. It was supplemented with 2 mmol urea plant^-1 ^during first two weeks, 1 mmol urea plant^-1^during the next two weeks and no more urea during the last two weeks. The nutrient solution was constantly aerated at a flow of 400 ml plant^-1 ^min^-1^. The pH was buffered close to 7 with CaCO_3 _(1 g l^-1^).

### Assessment of AMF colonization

At harvest, half of the root systems were used for estimation of the extent of root colonization by the AMF as follows: roots were cleared in KOH 10% at 80°C for 0.5 h followed by rinsing with water and two rinses with 1% HCl during 1 h. Thereafter, the roots were immersed at 80°C for 1.5 h in the staining solution consisting of lactic acid:glycerol:water (1:1:1 v:v:v) and 0.1% of each Trypan Blue and Methylene Blue.

After washing away the staining solution the roots were de-stained in tap water for 30 min at room temperature. The roots were examined under a compound microscope for quantitative colonization assessment by magnified-intersection method according to McGonigle et al. [17].

### Biomass and P content at harvest

The plants were harvested after 6 weeks of growth. Shoot, nodules and roots were separated and dried at 70°C for 48 h, and dry weight of each fraction was calculated.

The concentration of P was measured in samples of ground tissues following wet digestion with nitric-perchloric acids (6:1, v:v) at 250°C for 6 h, using the phosphovanado-molybdate method [18]. The P use efficiency (PUE) was calculated as the ratio of biomass (shoot + root) g^-1^/mean plant P content mg^-1^.

## Results

### Mycorrhization in hydroaeroponic-*versus *sand- cultures

**Figure 1:**
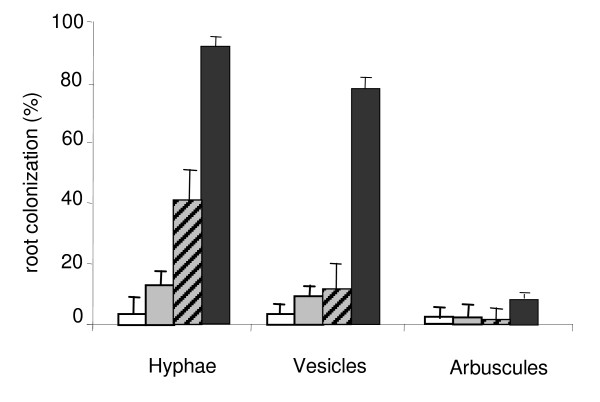
**Effect of *Glomus *on common bean root colonization by arbuscular mycorrhizal fungi**. Common bean genotype 115 grown in hydroaeroponic culture under P sufficiency (open bars) or P deficiency (grey bars) after inoculation with *Glomus *by mycorrhizal inoculant or by contact with mycorrhized *Stylosanthes guianensis *(hatched bars) both in sand pre-culture, and in sand culture (black bars). Data are means ± SD of means of three replicates plants harvested at 50 days after sowing.

Data in figure 1 show that the root-colonization was decreased by P supply since the rates of colonization by hyphea and vesicles were respectively 3.8 and 2.5 fold higher under P deficiency than under P sufficiency, though there was no significant difference in arbuscular colonization. Nevertheless higher colonization-rates were observed in sand culture, either after contact with mycorrhized *S. guianensis*, or after inoculation.

In order to assess the interaction of common bean with AMF diversity, the effects of three fungi spp were compared in hydroaeroponic culture and in sand with recombinant inbred line RIL115 (1^st ^experiment). All parameters of root-colonization were affected by both AMF identity, and the interaction with cultivation system (Table 1). Thus in hydroaeroponic culture, mycorrhization was found only with *Glomus *(Table 2). In sand culture, higher colonization-rates of hyphae and vesicles were found with *Glomus *than with *Gigaspora*. Regardless of the cultivation system, no colonization was found with *Acaulospora*.

**Table 1:** Influence of cultivation system and AMF species on percentage of common bean (RIL115) roots colonized by AMF hyphae (H%), arbuscules (A%) and vesicles (V%).

Colonization structure	Cultivation system (C)	AMF species (S)	C × S
H%	^$^14.49***	52.36***	6.00**
A%	4.22*	17.29***	3.13*
V%	11.00**	11.00***	19.15***

^$^F-ratios from two-way ANOVAs are shown with accompanying measures of statistical significance. * 0.01 ≤ *p *< 0.05; ** 0.001 ≤ *p *< 0.01; *** *p *< 0.001

**Table 2:** Influence of AMF species identity used for inoculation, on percentage of common bean (RIL115) roots colonized by AMF hyphae (H%), arbuscules (A%) and vesicles (V%) in various cultivation systems.

Cultivation system	Colonization structure	*Glomus*	*Gigaspora*	*Acaulospora*
Sand	H%	55.33 a	15.00 b	0.00 c
	V%	16.00 a	1.33 b	0.00 b
	A%	0.00 a	5.66 a	0.00 a
				
Hydroaeroponic 75 μmol P	H%	14.00 a	0.00 b	0.00 b
	V%	7.00 a	0.00 a	0.00 a
	A%	2.66 a	0.00 a	0.00 a
				
Hydroaeroponic 250 μmol P	H%	32.00 a	0.00 b	0.00 b
	V%	15.00 a	0.00 b	0.00 b
	A%	8.00 a	0.00 a	0.00 a

Different letters indicate significant differences between treatment means in one row.

### Interaction with plant genotype

In order to assess whether the tripartite symbiosis was affected by the common bean genotype, Flamingo and 4 RILs of the cross of BAT477 by DOR364, in addition to RIL115, were inoculated with *Glomus *and rhizobia (2^nd ^experiment). All parameters of root-colonization were affected by both genotype and cultivation system, and the interaction of the two factors (Table 3).

**Table 3:** Effect of cultivation system and genotypes on the extent of root colonization by hyphae(H%), arbuscules (A%) and vesicles (V%) of *Glomus *in common bean (115, 147, 83, 7, 28 and Flamingo).

Colonization structure	Cultivation system (C)	genotypes (G)	C × G
H%	^$^1011.33***	3.11*	2.20*
A%	68.89***	2.68*	1.96*
V%	1207.61***	4.04**	2.19*

^$^F-ratios from two-way ANOVAs are shown with statistical significance. * 0.01 ≤ *p *< 0.05;

** 0.001 ≤ *p *< 0.01; ***p < 0.001.

For hyphea in hydroaeroponic culture under P sufficiency, Flamingo and all RILs, except RIL28, had significantly higher root-colonization than RIL115, the highest difference being 3 fold for RIL147 (Table 4). Apart for RIL28 and Flamingo, P deficiency increased the hyphae colonization, this effect being the most significant for RILl47 (Table 4). In sand, all genotypes show higher rates of hyphal colonization than in hydroaeroponic cultures, this increase being the highest for RIL115 (Table 4).

For vesicles, in hydroaeroponic culture, the colonization was lower under P sufficiency than under P deficiency on RILs 115 and 83 (Table 4). However genotypic differences were found only in sand culture, where root-colonization was the highest, and where RIL115 classified the first among other genotypes (Table 4). Similarly for arbuscules, the root-colonization was highest in plants grown in sand followed by plants in hydroaeroponic culture under P deficiency (Table 4). Under P sufficiency most interestingly, no arbuscule was detected for RIls 83 and 7 (Table 4).

**Table 4:** Effect of *Glomus *on extent of root colonization by hyphae (H%), vesicles (V%) and arbuscules (A%) in common bean 115, 147, 83, 7, 28 and Flamingo, in hydroaeroponic culture under P sufficiency *versus *P deficiency and in sand culture.

		Cultivation system		
		
genotypes	Colonization structure	Sand	Hydroaeroponic 75 μmol P	Hydroaeroponic 250 μmol P
115	H%	91.33a	12.66a	3.33b
	V%	77.33ab	8.33a	3.33b
	A%	8.66a	1.33ab	1.33a
				
147	H%	86.66ab	20.00a	10.00a
	V%	76.00ab	11.33a	8.66a
	A%	8.66a	1.33ab	0.66a
				
83	H%	76.66bc	12.00a	9.33a
	V%	68.66bc	8.00a	4.00b
	A%	6.66a	1.33ab	0.00a
				
7	H%	75.33bc	15.33a	8.00ab
	V%	62.00c	10.00a	6.00ab
	A%	7.33a	0.66b	0.00a
				
28	H%	73.33c	10.66a	4.00b
	V%	68.66bc	11.33a	7.33a
	A%	6.66a	4.66a	1.33a
				
Flamingo	H%	86.66ab	10.66a	7.33ab
	V%	80.66a	13.33a	8.00a
	A%	10.66a	3.33ab	1.33a

Data are means of three replicates, for each structure, different letters indicate significant differences between treatment means in one column.

### Relation with nodulation

**Figure 2:**
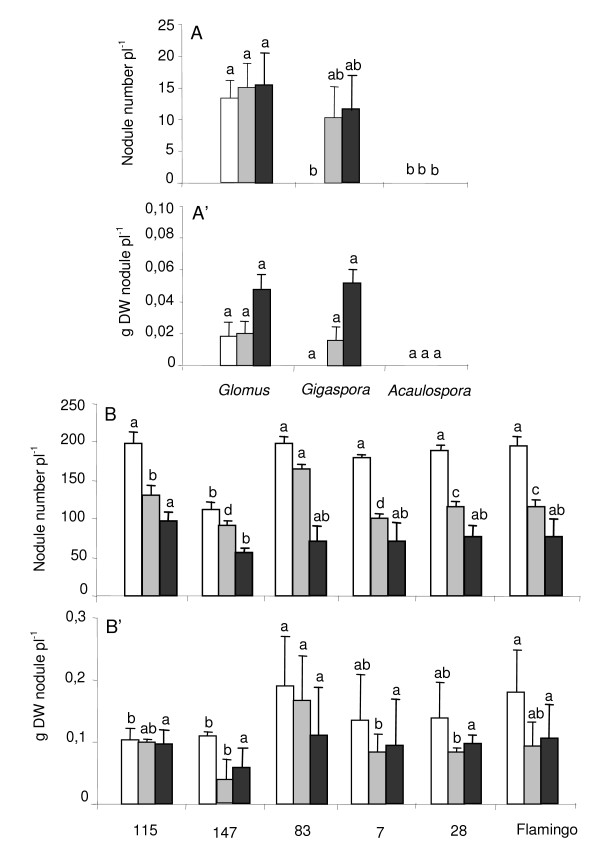
**Effect of *Glomus*, *Gigaspora *and *Acaulospora *on common bean number and dry weight of nodules**. Common bean genotype 115 (A and A') inoculated with *Glomus*, *Gigaspora *and *Acaulospora*, and recombinant inbred genotypes 115, 147, 83, 7, 28 and Flamingo (B and B') inoculated with *Glomus*, grown in hydroaeroponic culture under P sufficiency (open bars) *versus *P deficiency (grey bars) or in sand culture (black bars). Data are means ± SD of three replicates harvested at 50 days after sowing. For each cultivation system, different letters indicate significant differences between treatment means.

Nodule number was affected by cultivation system and AMF species, and the interaction of the two main factors in the 1^st ^experiment with RIL115 only (*p *< 0.001, *p *< 0.001 and *p *= 0.016 respectively), and by cultivation system and genotype, and the interaction of the two main factors (*p *< 0.001) in the 2^nd ^experiment with *Glomus *only. In the 1^st ^experiment there was significantly more nodules per plant with *Glomus *than with *Gigaspora *whatever the culture system (Fig. 2A). In hydroaeroponics with *Glomus *the number of nodules per plant was higher under P deficiency than under P sufficiency (Fig. 2A). However it was the highest in sand (Fig. 2A). There was no nodule after inoculation with *Gigaspora *under P sufficiency, and with *Acaulospora *whatever the cultivation system and the P supply (Fig. 2A). Neither the cultivation system nor the inoculation with AMF, nor the interaction between both factors, affected the nodule mass per plant (*p *= 0.17) when nodulation occurred. There was no significant difference of nodule mass between AMF species whatever cultivation system (Fig. 2A').

In the 2^nd ^experiment significantly more nodules per plant were encountered in the hydroaeroponic culture under P sufficiency than P deficiency, the lowest nodulation being in sand culture (Fig. 2B). In both cultivation systems, the lowest nodule number was observed with RIL147 (Fig. 2B). Among other RILs, no significant difference in nodule number was observed in the hydroaeroponic culture under P sufficiency. Very contrastingly under P deficiency, the nodule number decreased with higher *Glomus *root-colonization whatever the genotype. This same result was also observed in sand.

Nodule mass per plant in hydroaeroponic culture under P sufficiency was significantly lower for RILs115 and 147 than for other RILs and Flamingo (Fig. 2B). By contrast under P deficiency, the nodule mass per plant was twice higher for RIL115 than RIL147, and the highest value was observed for RIL83. In sand culture, there was no significant difference in nodule mass per plant among genotypes except for RIL147 showing the lowest value (Fig. 2B').

### Relation with growth

**Figure 3:**
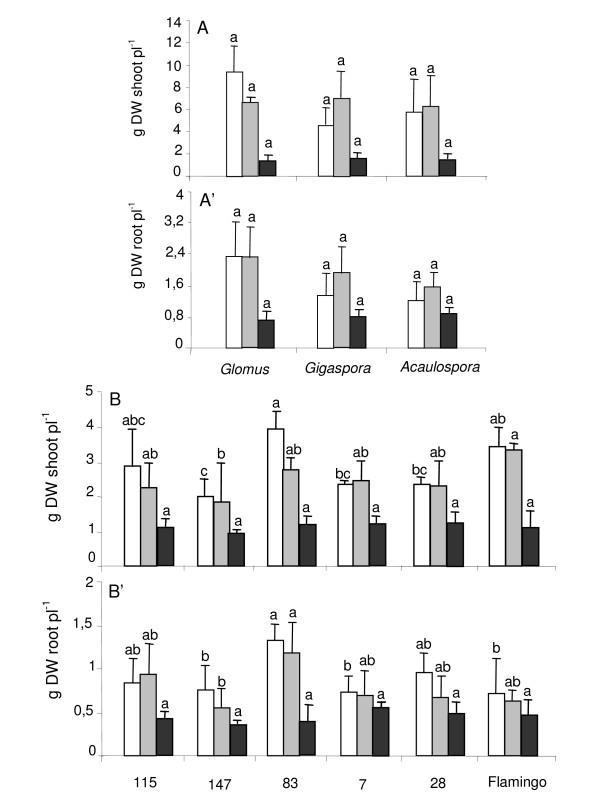
**Effect of *Glomus*, *Gigaspora *and *Acaulospora *on dry weight of shoot and root of common bean**. Common bean genotype 115 (A and A') inoculated with *Glomus*, *Gigaspora *and *Acaulospora*, and recombinant inbred genotypes 115, 147, 83, 7, 28 and Flamingo (B and B') inoculated with *Glomus*, grown in hydroaeroponic culture under P sufficiency (open bars) *versus *P deficiency (grey bars) or in sand culture (black bars). For each cultivation system, different letters indicate significant differences between treatment means.

In the 1^st ^experiment, shoot dry weight of RIL115 was significantly affected by the cultivation system (*p *< 0.001), and the interaction of the two main factors (system culture and AMF species, *p *= 0.04), but the AMF species had no significant effect (*p *= 0.11). Root dry weight was significantly affected only by cultivation system (*p *= 0.02), but not by AMF species (*p *= 0.25), neither by the interaction between the cultivation system and AMF species (*p *= 0.69). Systematically lower shoot and root dry weight of plants was observed in sand culture than in the hydroaeroponic culture (Fig. 3A &3A'). In the later, there was no significant difference in shoot and root dry weight of plants between deficient and sufficient P supplies. Regardless of the cultivation system, there was no significant effect of AMF species on shoot and root dry weight of plants.

In the 2^nd ^experiment, growth of plants was significantly affected by the cultivation system (*p *< 0.001) and the genotype (*p *< 0.001), but the interaction of the two main factors was not significant (*p *= 0.14). Systematically higher dry weight of plants was observed in hydroaeroponics than in sand (Fig.3B). Except for RIL83, there was no significant difference in shoot and root dry weight between deficient and sufficient P supplies in hydroaeroponics (Fig. 3B &3B'). There was no significant difference between RIL115 and other genotypes in sand culture for shoot and root dry weight (Fig. 3B &3B').

### Relation with P accumulation and P utilization efficiency

**Figure 4:**
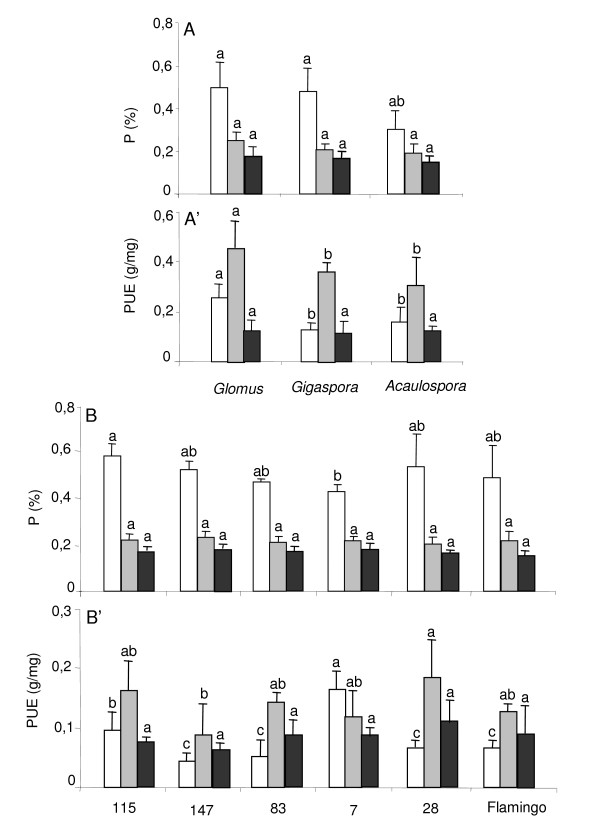
**Effect of *Glomus*, *Gigaspora *and *Acaulospora *on: shoot phosphorus content (%) and phosphorus use efficiency of common bean**. Common bean genotype 115 (A and A') inoculated with *Glomus*, *Gigaspora *and *Acaulospora*, and recombinant inbred genotypes 115, 147, 83, 7, 28 and Flamingo (B and B') inoculated with *Glomus*, grown in hydroaeroponic culture under P sufficiency (open bars) *versus *P deficiency (grey bars) or in sand culture (black bars). Data are means ± SD of three replicates harvested at 50 days after sowing. For each cultivation system, different letters indicate significant differences between treatment means.

Phosphorus content of the shoot of RIL115 was affected by the cultivation system (*p *< 0.001), but not by AMF species identity (*p *= 0.23) nor by the interaction of the two factors (*p *= 0.48) in the 1^st ^experiment. Phosphorus content in hydroaeroponics under P sufficiency compared to sand was nearly 3 fold higher with *Glomus *(0.50% ± 0.12 *versus *0.17% ± 0.02), 2.7 fold higher with *Gigaspora *(0.48% ± 0.11 *versus *0.17% ± 0.02) and 2 fold higher with *Acaulospora *(0.30% ± 0.08 *versus *0.14% ± 0.04) (Fig. 4A). Under P deficiency the phosphorus content was reduced by half with *Glomus *and *Gigaspora *(Fig. 4A). As a consequence, the P use efficiency (PUE) was strongly affected by the cultivation system (*p *< 0.001) and by AMF species identity (*p *< 0.05) but no by the interaction of the two factors (*p *= 0.61). The P use efficiency was 2 and 3 fold higher in hydroaeroponics under P deficiency than in sand, and higher with *Glomus *(0.45 ± 0.12 g DW mg^-1 ^P) than with *Gigaspora *or *Acaulospora *(0.36 ± 0.04 or 0.30 ± 0.11 g DW mg^-1 ^P) under P deficiency (Fig. 4A').

In the 2^nd ^experiment, the shoot phosphorus content was affected only by the cultivation system (*p *< 0.001), but not by genotypes (*p *= 0.20) nor by the interaction of these factors (*p *= 0.14). It was 3 and 3.5 fold higher in the hydroaeroponic culture under P sufficiency than under P deficiency or in sand for all genotypes (Fig. 4B). The P use efficiency was strongly affected by the cultivation system (*p *< 0.001), the genotypes (*p *= 0.005) and the interaction of the two factors (*p *= 0.014), being the highest for RIL7 (0.17 g DW mg^-1 ^P) and the lowest for RIL147 (0.04 g DW mg^-1 ^P) in the hydroaeroponic culture under P sufficiency (Fig. 4B').

## Discussion

It was possible in this work to establish the tripartite symbiosis of common bean with rhizobia and AMF under hydroaeroponic culture (Fig. 1), although the success varied between AMF species (Table 2). To our knowledge the hydroaeroponic culture is used with such intense aeration for the first time with the tripartite symbiosis with legumes, on the basis its previous use for rhizobial inoculation and N_2_-dependent growth with *Vigna *spp. [19], soybean [20], common bean [21] and *Acacia *spp. [22]. Indeed, the large majority of arbuscular mycorrhizal culture systems use nutrient solution with a solid career such as sand or glass beads [23,24]. Nevertheless, aeroponic culture of legumes colonized by both rhizobia and AMF have been recently reported [25,26], though it appears suitable only for very few symbiotic microorganisms such as *Glomus intraradices *[27]. The few other cultures of mycorrhized-plants without solid support were a follow up of pre-culture in solid career [28][29][30] like in our present study.

The lower colonization rates of roots in hydroaeroponics than in sand (Table 2 and 4) agrees with the previous observation of slower colonization of *Linum *by *Glomus intraradices *in hydroponic- than in sand- culture [29]. The difference in colonization between sand- and hydroaeroponic- culture could be explained by the orthosphosphate (Pi) supplied in the hydroaeroponic culture, since Pi is known to inhibit AMF colonization [31]. This could be due either to direct limitation by Pi in the solution, or to indirect limitation due to better P status of the plants grown in hydroaeroponics. Other limiting factors could be the lack of external AMF inoculum after transfer to hydroaeroponics, and the mechanical effects of the intense solution bubling. Thus, *Gigaspora *requires spores and mechanical stability for successful initiation and development of root colonization [32,33]. The dilution of signals involved in communication between the plant and the AMF could be another cause of lower mycorrhization in hydroaeroponic culture, and explain the differences between AMF species (Table 2). The absence of detectable root-colonization by *Acaulospora *could be due to incompatibility with common beans, or difficult staining of the root-colonization structures [34], or that staining of *Acaulospora *root colonization is tricky, it may be a false negative, since some *Acaulosporas *are difficult to stain with the blue dye [35].

The higher plant-growth in hydroponics than in sand suggests some nutrient limitation in sand where biomass of shoot and root were lower than in hydroaeroponics (Fig. 3). The most P limited conditions might have been in sand where the expression of mycorrhizal benefits was the most obvious (Table 2). This would agree with previous studies showing highest mycorrhizal benefits to plant growth under moderate P deficiency, especially with leguminous plants harbouring a coarser root system with less extension of root hairs than graminaceous [36,37]. The higher and regular P supply throughout the entire cultivation period in hydroaeroponics may have prevented the known mycorrhizal delivery of P from zones beyond the root P depletion zone [38]. Thus, the critical P supply for mycorrhizal benefit to plant growth remains to be established in hydroaeroponics, probably below 75 μml P since there was no significant difference of growth between 75 and 250 μml P treatments (Fig. 3).

The higher nodulation with *Glomus *than with the other AMF species in sand culture (Fig. 2A) is most likely due to improved P nutrition of the plants by *Glomus *that is known to be very efficient in transporting large quantities of P from remote zones to the plant [39]. This would in turn lead to allocation of larger amounts of P to the roots, promoting nodulation and potentially also the N_2 _fixation since many legumes are known to largely depend upon mycorrhizal P uptake [40,13]. By contrast, *Gigaspora *is known to supply lower quantities of P to the plants, usually with a delay caused by synthesis of polyphosphates in the extraradical mycelium [41]. This may also explain the negative effect in hydroaeroponics by diverting P from plant supply. Moreover, *Gigaspora *is known to require large quantities of C from the plant during the colonization establishment, sometimes leading to growth depressions of the host-plants [42]. These excessive carbon requirements by the AMF might prevent nodulation due to low availability of sugars in the roots, especially under conditions of suboptimal light supply [43] though the later was not the case of our study. There was much less effect of plant diversity on the symbiotic effects of *Glomus*.

## Conclusion

The successful establishment of mycorrhizal symbiosis in hydroaeroponic culture reported in this study opens possibilities for production of particularly clean material and *in situ *non-destructive studies of (i) signaling between the plants and their symbiotic fungi and the relation with rhizobial signaling for legumes, (ii) energetic balance in terms of carbon and oxygen requirements for symbiotic respiration, (iii) metabolic monitoring (NMR), and (iv) molecular analyses with in-situ hybridization [44] and RT-PCR. Together with other studies particularly addressing the spatial nutrient acquisition patterns and dynamics in the legume rhizosphere, we could soon achieve a level of mathematical modeling of P acquisition by a multispecies symbiotic complex [45].

## Authors' contributions

FT carried out the glasshouse experiment, measured the mycorhizal infection and drafted the manuscript. PS analyzed the biomass parameters. HV supplied the rhizobial inoculant. JJ supplied the mycorhizal inoculant and performed the statistical analysis. JJD conceived the study, and participated in its design and coordination. All authors read and approved the final manuscript.
